# Modulation of NRF2/KEAP1 Signaling in Preeclampsia

**DOI:** 10.3390/cells12111545

**Published:** 2023-06-04

**Authors:** Giovanni Tossetta, Sonia Fantone, Federica Piani, Caterina Crescimanno, Andrea Ciavattini, Stefano Raffaele Giannubilo, Daniela Marzioni

**Affiliations:** 1 Department of Experimental and Clinical Medicine, Università Politecnica delle Marche, 60126 Ancona, Italy; s.fantone@pm.univpm.it (S.F.); d.marzioni@univpm.it (D.M.); 2 Cardiovascular Internal Medicine, IRCCS Azienda Ospedaliero-Universitaria di Bologna, 40128 Bologna, Italy; federica.piani2@unibo.it; 3 Department of Medical and Surgical Sciences, University of Bologna, 40138 Bologna, Italy; 4 School of Human and Social Science, University “Kore” of Enna, 94100 Enna, Italy; caterina.crescimanno@unikore.it; 5 Clinic of Obstetrics and Gynaecology, Department of Clinical Sciences, Università Politecnica delle Marche, Salesi Hospital, 60123 Ancona, Italy; a.ciavattini@univpm.it (A.C.); s.r.giannubilo@staff.univpm.it (S.R.G.)

**Keywords:** NRF2, antioxidants, preeclampsia, pregnancy, pregnancy complications, NRF2/KEAP1, KEAP1, compounds, natural

## Abstract

Placentation is a key and tightly regulated process that ensures the normal development of the placenta and fetal growth. Preeclampsia (PE) is a hypertensive pregnancy-related disorder involving about 5–8% of all pregnancies and clinically characterized by de novo maternal hypertension and proteinuria. In addition, PE pregnancies are also characterized by increased oxidative stress and inflammation. The NRF2/KEAP1 signaling pathway plays an important role in protecting cells against oxidative damage due to increased reactive oxygen species (ROS) levels. ROS activate NRF2, allowing its binding to the antioxidant response element (ARE) region present in the promoter of several antioxidant genes such as heme oxygenase, catalase, glutathione peroxidase and superoxide dismutase that neutralize ROS, protecting cells against oxidative stress damages. In this review, we analyze the current literature regarding the role of the NRF2/KEAP1 pathway in preeclamptic pregnancies, discussing the main cellular modulators of this pathway. Moreover, we also discuss the main natural and synthetic compounds that can regulate this pathway in in vivo and in vitro models.

## 1. Introduction

The placenta is an essential organ that deeply changes in morphology and function during pregnancy, performing important functions during pregnancy and ensuring the normal fetal development [1,2]. Preeclampsia (PE) is a hypertensive pregnancy-related disorder involving about 5–8% of all pregnancies. It generally appears from the second trimester of gestation and it is clinically characterized by de novo maternal hypertension (a diastolic blood pressure of ≥90 mmHg and/or systolic blood pressure of ≥140 mmHg) and proteinuria (>300 mg/24 h). A high BMI, previous preeclamptic pregnancy, advanced maternal age, nulliparity and severe COVID-19 are important risk factors of PE [3,4,5]. In the case of severe PE, there is also the risk for the mother to develop comorbidities such as eclampsia, hepatic alterations (hemolysis, elevated liver enzymes and low platelets (HELLP) syndrome), renal failure and disseminated vascular coagulation (DIC). PE may also lead to further complications for the fetus, causing fetal growth restriction (FGR), prematurity and fetal death [6,7].

Although there have been many progresses in understanding the pathophysiology of this disease over the past decade, many of the key mechanisms characterizing PE are still unknown. In particular, it has been shown that an impaired invasion of the extravillous trophoblasts (EVT) into the maternal uterine wall leads to a compromised remodeling of the spiral uterine arteries, leading to a not physiological hypoxic condition during pregnancy [7], causing trophoblast immaturity [8] and an altered angiogenesis [9] of placental villi. This hypoxic condition is a favorable environment for the production of free radicals, leading to oxidative stress and inflammation [10], a key process also involved in other pregnancy complications such as preterm delivery [11,12] and gestational diabetes mellitus (GDM) [13].

Oxidative stress is involved in several pathological conditions including infertility [14,15], ocular diseases [16], pregnancy complications [17], endothelial dysfunction [18,19], osteoporosis [20], neuronal diseases [21] and cancer [22,23,24,25,26,27]. Generally, all cells of the organism are exposed to oxidative stress but during pregnancy there is a high fetal and maternal oxygen request that leads to an increase in reactive oxygen species (ROS), superoxide radicals (O^2−^), hydrogen peroxide (H_2_O_2_), the hydroxyl radical (OH^−^) and singlet oxygen production with consequent damaging of placental cells [28]. Although cells are provided of antioxidant enzymes such as superoxide dismutase (SOD), glutathione peroxidase (GSH-Px), catalase (CAT) and glutathione (GSH), to prevent cellular damage from ROS, their activity is reduced in PE pregnancies [29]. This could be due to the presence of genetic variations such as single nucleotide polymorphisms (SNPs) in the catalytic site of the enzymes that may significantly impair their enzymatic activity, making them less efficient in counteracting ROS. This makes placental and maternal cells more vulnerable to ROS damage, increasing the risk of PE onset during pregnancy [30,31].

The Nuclear Factor Erythroid 2-Related Factor 2 (NFE2L2 or NRF2)/Kelch-like ECH-associated protein 1 (KEAP1) signaling regulates gene expression of a wide variety of cytoprotective antioxidant and phase II detoxification enzymes. KEAP1 is a cytoplasmic protein that binds to NRF2 and promotes its degradation by the proteasome. CULLIN 3 (CUL3) is a scaffold protein that forms a complex with KEAP1 and NRF2. This complex is responsible for the ubiquitination and degradation of NRF2 under basal conditions. Under oxidant stimuli, ROS bind the cysteine residues of KEAP1, causing a conformation change in KEAP1 that inhibits NRF2 ubiquitination and allows NRF2 translocation into the nucleus to bind the antioxidant response element (ARE) regions present in the promoter of antioxidant genes, inducing their transcription [22,32,33]. Thus, NRF2 signaling can modulate many antioxidant enzymes impaired in PE, reducing inflammation [34]; it follows that a proper modulation of the NRF2 signaling pathway could ameliorate many placental disfunctions found in this pathology, improving the pregnancy outcome.

NRF2 can be activated by several compounds by modulating its interactions with KEAP1 or by promoting its nuclear translocation (e.g., sulforaphane, curcumin and resveratrol) [23,35] while other compounds have showed inhibitory effects on NRF2 by directly targeting NRF2 or its downstream effectors (e.g., brusatol, trigonelline and ML385) [36,37,38].

Here, we review and discuss the literature regarding the main cellular modulators of the NRF2/KEAP1 signaling pathway in preeclamptic pregnancies. Moreover, we report the main natural and synthetic compounds that can regulate this pathway in in vivo and in vitro models.

## 2. NRF2/KEAP1 Signaling in PE

It has been reported that low amounts of ROS may be necessary to maintain normal cellular signaling, acting as important intracellular messengers, while high ROS levels are associated with cell toxicity [39,40]. This “protective” effect of ROS could also be involved in placental angiogenesis, a critical process impaired in PE [41,42].

The clinical impact of NRF2/KEAP1 signaling in PE has been deeply discussed by Kweider and colleagues [43]. It is known that PE patients typically have a low antioxidant capacity but increased placental oxidative stress [44]. Thus, NRF2/KEAP1 signaling may exert beneficial effects by preventing/attenuating PE symptoms. It is also known that ROS can increase inflammatory cytokine levels, inducing trophoblast apoptosis and favoring hypertension by impairing the vasodilator and contractile responses of vascular smooth muscle [45]. The importance of NRF2/KEAP1 signaling in PE is further highlighted by the fact that many downstream genes of this pathway (e.g., SOD3, NQO1, catalase and HO-1) have showed protective effects against hypertension [46,47,48,49]. However, it deserves to be pointed out that no difference in blood pressure has been found between NRF2-knockout and wild-type mice, suggesting that hypertension is modulated by a more complex mechanism, where NRF2 plays a minor role [50].

An interesting study showed that knocking down NRF2 in pregnancy-associated hypertension (PAH) mice (NRF2^−/−^ PAH mice), a mouse model of PE, improved maternal and fetal survival, ameliorated intra-uterine growth retardation and increased oxidative DNA damage. Furthermore, placentas of these mice showed many characteristics of human preeclampsia including an increased endothelial cell proliferation and a dense vascular network. The authors also found that NRF2 deficiency in these mice induced the mRNA expression of angiogenic chemokines such as Ccl2 (encoding C-C motif chemokine ligand 2), Ccl5 (encoding C-C motif chemokine ligand 5), Cxcl9 (encoding C-X-C motif chemokine ligand 9) and Cxcl10 (encoding C-X-C motif chemokine ligand 10). Moreover, there was a significant increase in mRNAs encoding inflammation-related cytokines such as interleukin (IL)-1α, IL-1β, tumor necrosis factor (TNF)-α and C-X-C motif chemokine ligand 1. The authors also reported an increase in ROS levels in labyrinth placentas of NRF2^−/−^ PAH mice accompanied by a low NAD(P)H dehydrogenase quinone 1 (NQO1) and sulfiredoxin 1 (SRXN1) expression, two NRF2 downstream genes, in these placentas. Thus, ROS-mediated signaling plays a key role in maintaining placental angiogenesis in PE [51].

Utero-placental interaction plays a key role in the maternal–fetal crosstalk during pregnancy and abnormal uterine vascular remodeling has been associated with PE and FGR increasing the risk of a preterm birth [52,53]. It has been found that the oxidative stress level was significantly increased but no changes were found in decidual NRF2 and KEAP1 protein expression in the decidua of PE pregnancies complicated by FGR. Interestingly, PE pregnancies with normal fetal growth also showed increased decidual oxidative stress but NRF2 expression was reduced and KEAP1 expression was increased in uterine areas of high trophoblast density, suggesting that decidual oxidative stress is modulated by trophoblast cells. This is an important study that underlines the different pathophysiology of PE with and without FGR [54]. Impaired alteration of the NRF2 pathway in PE compared to normal pregnancies was also confirmed by using genome-wide transcriptional profiling of the decidua basalis. In fact, Løset and colleagues found an increased expression of genes belonging to the NRF2/KEAP1 signaling pathway among the genes differently expressed in the decidua from PE pregnancies [55]. Furthermore, Kweider and colleagues found that NRF2 immunostaining was significantly increased in endovascular and interstitial trophoblastic cells of the placental bed and in the extravillous trophoblast of pregnancies complicated by early-onset PE-FGR compared to normal ones [56].

In addition, it has been reported that NRF2 expression significantly decreases in the syncytiotrophoblast, villous stromal cells and vascular endothelium in placentas of PE pregnancies [57]. In contrast to this study, Wruck and colleagues found that NRF2 expression was exclusively expressed in the villous cytotrophoblast. Moreover, they found that NRF2 expression in cytotrophoblastic nuclei of PE placentas was significantly higher than normal gestation-matched controls, suggesting an increased activation of this signaling in PE while the syncytiotrophoblast was negative in both controls and PE placentas. Thus, the expression of NRF2 within cytotrophoblastic cells strongly suggests that these cells are involved in the expression of NRF2-dependent antioxidant genes under oxidative stress [58].

It is known that ATP-binding cassette (ABC) transporters regulate substrate flow between maternal and fetal circulation [59], playing a key role in the antioxidant defense of the placenta by pumping out toxic oxidative metabolites [60]. Moreover, it has been reported that NRF2 can stimulate the expression of these transporters by binding the ARE regions present in their gene promoter [22,24,61]. Yu and colleagues demonstrated that the levels of NRF2, heme oxygenase-1 (HO-1), ABC transporter multidrug resistance-associated protein (MRP) 1, -2, breast cancer resistance protein (BCRP) and P-glycoprotein (Pgp) were significantly lower both in PE placentas compared to normal pregnancies and in the placentas of early-onset PE compared to late-onset PE. NRF2 silencing in JEG3 cells and NRF2 knockdown in mice significantly downregulated the expression of HO-1, MRP1, -2, BCRP and Pgp [62]. Thus, the impaired NRF2 expression in PE pregnancies can favor the increase in oxidative stress by altering the expression of ABC transporters.

## 3. NRF2 Cellular Modulators

Ferroptosis is a programmed cell death process involved in the alteration of iron metabolism, amino acids, GSH, ROS and lipid peroxides (LPOs) in the plasma membrane [63]. Thus, ferroptosis plays a key role in regulating cell fate under oxidative stimuli. DJ-1 is a small binding protein located on cell membranes that acts as a sensor for the redox state, causing NRF2 dissociation from KEAP1 and its translocation into the nucleus to activate the NRF2-responding genes [64]. It has been reported that the inhibition of DJ-1 can enhance the sensitivity of tumor cells to ferroptosis inducers and that DJ-1 depletion plays a role in promoting ferroptosis [65]. DJ-1 expression has been found to be significantly higher in PE placentas than normal pregnancies, suggesting that the overexpression of DJ-1 in PE placentas could act as a compensatory mechanism for hypoxia [66]. Interestingly, it has been found that the concentration of malondialdehyde (MDA), a ferroptosis marker, was significantly higher in the PE pregnancies compared to normal pregnancies. Moreover, NRF2, DJ-1 and glutathione peroxidase 4 (GPX4) expressions were significantly higher in PE pregnancies. Importantly, authors found the NRF2 and GPX4 expressions were significantly reduced when DJ-1 was knocked down in BeWo cells and when these cells were treated with RSL3, a ferroptosis inducer. Lactate dehydrogenase (LDH) release was significantly increased in DJ-1 knockdown compared to wild-type BeWo cells, indicating increased cell death. In this study, the authors showed that ferroptosis is involved in the pathogenesis of PE and that DJ-1 plays an important protective role in this pathology by mediating ferroptosis in trophoblast cells through the regulation of the NRF2/GPX4 signaling pathway [67].

Vascular endothelial growth factor (VEGF) has been reported to be decreased in PE and may be involved in the activation of the NRF2 pathway. In fact, an interesting study found that VEGF activated NRF2 in a BeWo cell line, leading to an increase in antioxidative enzymes such as thioredoxin (Trx), thioredoxin reductase (TXNRD1) and heme oxygenase-1 (HO-1). HO-1 metabolizes heme to generate biliverdin, iron and carbon monoxide (CO). The latter enhances VEGF synthesis in vascular smooth muscle, promoting its relaxation and vasodilatation [68]. Importantly, authors found that NRF2 activation by VEGF was ERK1/2 dependent since NRF2 activation was inhibited by ERK inhibitors. Interestingly, they found an antioxidant effect of VEGF via a positive feedback loop. In fact, VEGF activated NRF2 in an ERK1/2-dependent manner, increasing HO-1 expression and the production of carbon monoxide (due to the HO-1 activity), which in turn up-regulated VEGF expression. Thus, decreased VEGF bioavailability during PE may result in higher vulnerability to oxidative damage [69].

It has been found that serum concentration of oxidized low-density lipoprotein (oxLDL) is higher in women with PE [70,71]. oxLDL is removed from circulation by lectin-like oxLDL receptor-1 (LOX-1), which acts as a scavenger receptor for oxLDL [72]. It has been reported that oxLDL activates the NRF2/KEAP1 signaling pathway [73]. Interestingly, Chigusa and colleagues found that LOX-1, NRF2 and HO-1 expression was significantly decreased in PE placentas compared to normal pregnancies. Moreover, oxLDL treatment of JAR cells significantly increased NRF2 and HO-1 expression while the blockade of LOX-1 by TS92, an anti-human LOX-1 antibody, significantly inhibited the increase in HO-1 expression induced by oxLDL treatment. Thus, the decreased LOX-1 expression in PE may contribute to the high oxLDL concentration, low NRF2 activation and low HO-1 expression found in this pathology [74].

It has been reported that increased oxidative stress and decreased antioxidant capacity are among the main factors involved in endothelial cell hyperpermeability, a key event in the pathogenesis of PE [75]. Lipoxins are endogenous mediators derived from arachidonic acid that play an important role as anti-inflammatory compounds [76]. It has been found that lipoxin A4 (LXA4) deficiency is associated with PE-like symptom onset [77,78]. Moreover, Pang and colleagues found that LXA4 strongly attenuated lipopolysaccharide (LPS)-induced hyperpermeability in human umbilical vein endothelial cells (HUVEC) through maintaining the normal expression of VE-cadherin and β-catenin. Interestingly, LXA4 inhibited LPS-triggered ROS production, promoting the expression of NRF2 and demonstrating a key role of the LXA4/NRF2 axis in regulating vascular permeability under oxidant stimuli [79].

It is known that PE pregnancies are exposed to chronic hypoxia due to the shallow invasion of trophoblasts into the uterine wall that fails in maternal spiral artery remodeling [80,81]. It has been reported that hypoxia promoted the translocation of NRF2 into the nucleus in HTR-8/SVneo cells, leading to NRF2/HO-1 signaling activation. Moreover, hypoxia reduced the invasion of HTR-8/SVneo cells and induced oxidative stress, increasing malondialdehyde (MDA), ROS and induced ferroptosis [82]. Interestingly, NRF2 overexpression in hypoxia-induced HTR-8/SVneo cells reduced the levels of MDA and ROS, and decreased ferroptosis, proving that NRF2 signaling activation plays a protective role in PE [83]. Another study found that under hypoxic conditions, the activity of catalase (CAT), GSH-Px and SOD enzymes in HTR8/SVneo cells was significantly lower. Moreover, hypoxic conditions increased NRF2 and HO-1 expression while decreasing KEAP1 expression. In addition, the enzymatic activity of SOD, GSH-Px and CAT in placental tissues of patients with PE was significantly lower compared to normal placental tissues. Interestingly, NRF2 and HO-1 expression was significantly higher while KEAP1 expression was lower in PE placentas compared to normal placentas. NRF2 silencing significantly reduced the activities of CAT, GSH-Px and SOD in HTR8/SVneo cells under hypoxic conditions, proving an important antioxidant effect of the NRF2/KEAP1 signaling pathway in these cells [84]. The previous two studies were further confirmed by Feng and colleagues, reporting lower CAT, GSH-Px and SOD activity in HTR8/SVneo cells under hypoxic conditions and in PE placentas. Furthermore, the authors reported increased NRF2 and HO-1 expressions and reduced KEAP1 expression under hypoxic conditions and in PE placentas [85].

Advanced glycation end products (AGEs), advanced oxidation protein products (AOPPs) and advanced lipid peroxidation products (ALEs) are the three most common biomarkers used to evaluate protein modifications caused by oxidative stress [86,87]. AOPPs are a family of oxidized, dityrosine-containing products formed by plasma proteins and chlorinated oxidants’ interaction under oxidative stress commonly present in several diseases [88,89,90,91] including PE [92]. In fact, it has been found that AOPPs can increase oxidative stress, leading to trophoblast dysfunction and apoptosis, showing an important involvement of AOPPs in PE pathogenesis [92]. Interestingly, it has been found that AOPPs directly increased apoptotic protein expressions and significantly inhibited the expression of the NRF2/ARE/HO-1 pathway in HTR-8/SVneo cells. The authors found that NRF2 silencing significantly aggravated the AOPP-induced cell apoptosis, activating p53 and the caspase cascade while NRF2 overexpression had the opposite effect, leading to cytoprotective effects by increasing HO-1 expression in HTR-8/SVneo cells. Hence, NRF2/ARE/HO-1 pathway activation plays an important role in AOPP-induced cell apoptosis, suggesting this pathway as a therapeutic target against PE [93].

MicroRNAs (miRNAs) are short (around 20 nucleotides) non-coding RNA sequences involved in the regulations of several cellular processes in cancerous [94,95,96,97] and non-cancerous [98,99,100,101] diseases. MiR-133a has been reported to be involved in inhibiting cancer cell proliferation in various cancer types [102,103]. However, miR-133a also showed important protective functions in non-cancerous diseases such as hypoxia-induced oxidative stress in cardiomyocytes, reducing apoptosis [104]. Oxidative stress is also a characteristic feature of PE pregnancies and is the cause of many complications found in this pathology [31]. An interesting study found that transfecting HTR-8/SVneo cells with miR-133a-3p under an oxidative stress condition (induced by H_2_O_2_) relieved the oxidative stress induced by H_2_O_2_ through the reduction in ROS and MDA levels. Moreover, the authors found reduced oxidative stress-induced apoptosis. The authors proved that this antioxidant effect of miR-133a-3p was due to the inhibition of its target gene BTB domain and CNC homolog 1 (BACH1), a transcriptional repressor that competes with NRF2 for binding to ARE (antioxidant response element) sites on target genes. BACH1 can inhibit NRF2 activity by preventing its binding to ARE sites [105]. In fact, miR-133a-3p overexpression decreased BACH1 expression and increased NRF2 activation, leading to an increase in HO-1 expression [106]. Thus, miR-133a-3p can relieve the oxidative stress-induced apoptosis of trophoblast cells through the BACH1/NRF2/HO-1 signaling pathway. MiR-1246 is known to target Axin-2 and glycogen synthase kinase-3 β (GSK3β), two inhibitors of the Wnt/β-catenin pathway [107], which is involved in trophoblast proliferation and differentiation [108]. Another protein involved in trophoblast differentiation is the aromatase CYP19A1, which synthetizes estrogens, key hormones that regulate syncytiotrophoblast differentiation [109]. An interesting study by Muralimanoharan and colleagues found that NRF2, CYP191A mRNAs and miR-1246 levels were significantly upregulated in a primary culture of human trophoblast cells during syncytiotrophoblast differentiation and significantly reduced by hypoxia and in PE placentas. Moreover, the expression of Axin-2 and GSK3β was significantly downregulated during syncytiotrophoblast differentiation. The authors also found a downregulation of Jumonji and AT-rich interaction domain containing 2 (JARID2), a cell differentiation inhibitor [110]. NRF2 silencing in cytotrophoblast cells significantly inhibited miR-1246 and CYP19A1. Furthermore, NRF2 could bind miR-1246 and CYP191A promoters, explaining their increase during syncytiotrophoblast differentiation. Thus, NRF2 can promote syncytiotrophoblast differentiation by inducing JARID2 expression, and CYP19A1 and miR-1246, which inhibit the expression of Axin-2 and GSK3β [111].

CD151 is a member of tetraspanins [112] with antioxidant properties [113]. An interesting study by Wang and colleagues found that CD151 expression was significantly downregulated in PE pregnancies. Moreover, PE pregnancies showed a decreased expression of the antioxidant enzymes HO-1, NQO1, glutamate–cysteine ligase catalytic subunit (GCLC) and SOD-1. Overexpression of CD151 in HTR-8/SVneo cells enhanced HO-1, NQO1, GCLC and SOD-1 expression. These effects were reverted after silencing of CD151 in these cells. Interestingly, tail intravenous injection of siCD151 in pregnant mice led to a PE-like phenotype inducing hypertension and proteinuria. Moreover, the expression of NRF2, pERK1/2, HO-1, NQO-1, GCLC and SOD-1 was significantly decreased in mice and HTR8/SVneo when CD151 was silenced, suggesting an involvement of ERK signaling in CD151 antioxidant function. In fact, the beneficial effect of CD151 was significantly inhibited when ERK and NRF2 signaling were blocked with synthetic inhibitors (SCH7 72984 and ML385, respectively) in HTR8/SVneo cells. Thus, in trophoblast cells, CD151 exerts its antioxidant function trough NRF2 and ERK signaling pathways [114].

Bromodomains (BRDs) are functional domains present on the bromodomain and extraterminal (BET) family of proteins. The function of these domains is to activate gene transcription by recruiting polymerases and transcription factors. Among the BRD proteins, BRD4 protein has been studied as a regulator of oxidative stress [115]. BRD4 mRNA and protein expression increased after H_2_O_2_ exposure while BRD4 inhibition attenuated H_2_O_2_-induced oxidative stress injury in an HTR-8/SVneo cell line [116]. BRD4 knockdown increased cell proliferation and invasion while decreasing apoptosis and ROS production following H_2_O_2_ exposure. Moreover, suppression of BRD4 significantly decreased KEAP1 expression, but increased the nuclear expression of NRF2. The protective effect of BRD4 inhibition was reversed by KEAP1 overexpression or NRF2 inhibition. Thus, BRD4 inhibition attenuated oxidative stress injury by enhancing NRF2 activation via KEAP1 downregulation.

**Figure 1:**
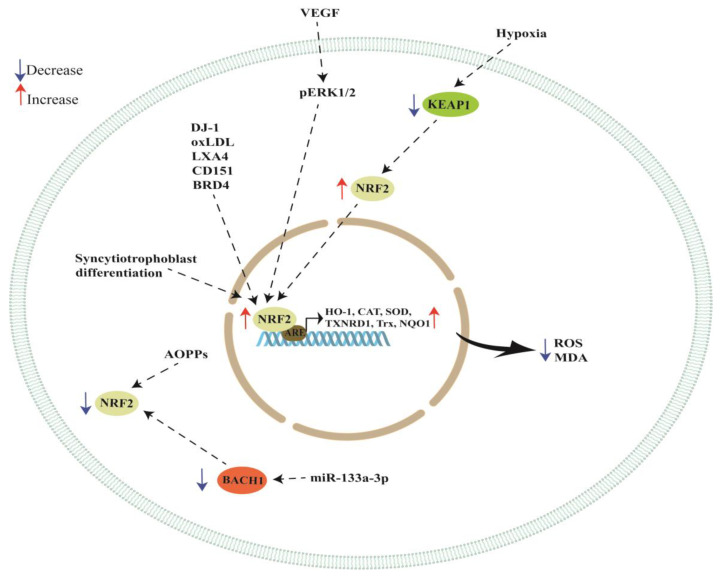
Schematic representation of NRF2 modulation by cellular regulators.

A schematic representation of NRF2/KEAP1 pathway regulation by cellular modulators is reported in Figure 1. Studies discussed in this section are summarized in Table 1.

## 4. NRF2 Modulation by Natural Compounds

Procyanidin B2, widely distributed in plants, exhibits antioxidative activity, mitigation of endoplasmic reticulum stress and anti-inflammatory effects [117]. In a rat model of PE, the increased soluble fms-like tyrosine kinase-1 (sFlt-1) levels were associated with decreased activity of peroxisome proliferator-activated receptor γ (PPARγ). PPARγ is a transcription factor involved in antioxidative stress, trophoblast differentiation, anti-inflammation and normal vascular function and it is induced by procyanidin B2 in primary placental tissues and endothelial cells. Since NRF2 has been reported to bind the PPARγ promoter region, enhancing its transcriptional activity, the NRF2/PPARγ signaling pathway is involved in regulating the inhibitory effect of procyanidin B2 on sFlt-1 secretion [118].

Resveratrol is a polyphenolic compound present in a variety of fruits, mostly in red grapes. In cultured endothelial cells, resveratrol up-regulates gene expression of the antioxidant defense enzymes NQO1 and HO-1 in an NRF2-dependent manner [119]. HUVEC incubated with plasma from women with PE showed increased antioxidant response element (ARE) activity and the addition of resveratrol was able to potentiate this effect. Oxidative stress is a stimulus that leads to the NRF2 activation, which may be enhanced by the addition of resveratrol in PE [120]. Thus, resveratrol could mitigate or reverse placental and endothelial cellular oxidative stress. In particular, NRF2 can reduce activin A, and OH-1 decreases sFlt-1 levels [121,122]. Interestingly, the experiments of Gurusinghe et al. [123] showed that resveratrol decreased vascular cell adhesion molecule 1 (VCAM-1) and endothelin-1 expression in endothelial cells while decreasing sFlt-1 and activin A in NRF2 knockdown placental cells.

Recently, various studies evaluated the antioxidant activity of natural compounds such as flavonoids in the activation of NRF2. Silibinin is a flavonolignan composed of a flavonoid and a phenylpropane isolated from *Silybum marianum*, which has been shown to exhibit antioxidant and antineoplastic activities. A study showed that silibinin may reduce apoptosis in extravillous trophoblast cell lines while recent studies suggested that silibinin enhanced the activation of NRF2 in HTR8/SVneo cell lines treated with H_2_O_2_ [124]. Among flavonoids, fisetin (3,3′,4′,7-tetrahydroxyflavone) is a natural flavonoid commonly found in many fruits and vegetables with a wide range of pharmacological functions including anti-inflammatory, antioxidant and anti-tumor activities [125]. The antioxidant activity has been demonstrated by increasing the transcriptional activity of NRF2 [126]. In preeclampsia-like rat models, fisetin reduced the clinical and biochemical setting of PE by activation of Toll-like receptor 4 (TLR4)/NF-κB and NRF2/HO-1 pathways [127].

Apocyanin and sulforaphane are natural compounds widely present in plant extracts with biological interest [22,128]. Apocyanin is an inhibitor of the NADPH–oxidase complex that acts on impairing membrane translocation of the cytosolic component p47phox (a member of the NADPH–oxidase complex) [129]. Combined treatment of apocyanin and aspirin ameliorated the PE symptoms by activating the phosphoinositide 3-kinase (PI3K)/NRF2/HO-1 pathway in rat models of PE [130]. Sulforaphane, an aliphatic isothiocyanate found in many cruciferous vegetables, is rapidly metabolized after catalyzation by glutathione S-transferase. Sulforaphane is believed to exert antioxidant effects by up-regulating NRF2 expression [32,131]. An interesting study evaluated the effect of sulforaphane in endothelial dysfunction, a characteristic of PE pregnancies. This study demonstrated that sulforaphane is able to protect endothelial cells and reduce placental secretion of vasoactive agents such as endothelin 1, intercellular adhesion molecule 1 (ICAM-1), vascular cell adhesion molecule 1 (VCAM-1) and E-selectin. In placental explants, sulforaphane reduced sFlt-1, soluble endoglin (sEnd) and activin A secretion. However, this effect was not blocked by NRF2 silencing, suggesting that the NRF2 signaling pathway is not involved in this mechanism [132].

Crocin is a hydrophilic carotenoid derived from saffron (or *Crocus sativus*) with beneficial effects on cardiovascular pathologies, obesity and hypertension [133]. In a PE rat model, the administration of crocin led to a significant reduction in blood pressure and placental growth factor (PlGF) and decreased sFlt-1 levels. Clinically, an increase in the fetal weight, fetal survival and fetal/placenta ratio was achieved. These phenotypic changes were accompanied by upregulation of protein levels of NRF2 and HO-1 [134]. Astragaloside IV, an extract from *Astragalus membranaceus*, has been studied primarily for its properties on the cardiovascular system through its action in protecting the endothelium from lipoprotein damage and hyperglycemia [135]. This compound has been studied for its anti-inflammatory action in liver, lung and cardiovascular disease [136]. In a PE rat model, administration of astragaloside IV, in a dose-dependent manner, significantly reduced sFlt-1 and PlGF levels as well as improved the systolic blood pressure, proteinuria, placental weight and overall fetal survival. These clinical improvements were accompanied by an overall reduction in placental cellular oxidative stress, inducing a partial recovery of NRF2 and HO-1 placental levels [137].

Pyrroloquinoline quinone is an essential animal nutrient with important functions on mammalian development, growth, reproduction and immune function [138]. Pyrroloquinoline quinone is an important antioxidant compound, which exerts its function through the protection of the mitochondria against oxidative stress-induced lipid peroxidation, protein carbonyl formation and inactivation of the mitochondrial respiratory chain [139]. In a PE rat model, dietary intake of pyrroloquinoline quinone significantly increased NRF2 expression [140]. Another powerful antioxidant, 1-O-hexyl-2,3,5-trimethylhydroquinone (HTHQ), a derivative of vitamin E, showed several positive effects on diabetes, hepatic cirrhosis, neurodegenerative diseases and cancer [141,142,143]. It has been reported that HTHQ treatment of a PE mouse model significantly induced nuclear translocation and expression of NRF2, leading to an increased HO-1 expression in placental tissues and providing antioxidant and antiapoptotic effects [144].

Coenzyme Q10 is a fat-soluble ubiquinone with intracellular antioxidant activity synthesized endogenously from phenylalanine (benzoquinone ring) and mevalonic acid (responsible for isoprenoid units) but it can also be provided (in a minor part) from the diet. In the mitochondrial respiratory chain, it is responsible for electron transport from complex I and II to complex III [145,146]. Coenzyme Q10 was found to play an important role as an antioxidant in plasma lipoproteins, where it also regenerates the active form of vitamin E. When orally administered to mice [147] and rabbits [148], coenzyme Q10 showed a significant anti-atherosclerotic effect. It has been reported that coenzyme Q10 levels were significantly decreased in PE pregnancies [149]. Coenzyme Q10 treatment induced NRF2 and HO-1 up-regulation in the placental tissues of PE rat models (both nuclear and total NRF2 protein levels) [150].

Melatonin, a lipid-soluble hormone with antioxidant activities, is primarily produced by the pineal gland but it is also produced in large quantities by the placenta and it is rapidly transferred from maternal to fetal circulation [151]. This hormone regulates the circadian rhythm but also shows direct free radical scavenging properties and can induce the expression of antioxidant enzymes [152]. The melatonin levels are significantly reduced in PE, as well as decreased levels of the enzymes responsible for melatonin synthesis and decreased levels of melatonin receptors [153]. In animal models (sows), dietary supplementation with melatonin was associated with a significant increase in placental and newborn weight and with a significant increase in placental mRNA levels of antioxidant-related genes involved in the NRF2/ARE pathway (NRF2, SOD, GPx1 and NQO1) [154]. When administrated intraperitoneally to a PE rat model, melatonin treatment decreased blood pressure and proteinuria. These effects were accompanied by an increased serum sFlt-1/PlGF ratio and increased levels of NRF2, PlGF and HO-1 in the placenta [155]. These effects have been found to be comparable to the use of aspirin, a drug that significantly reduces PE onset and mortality [156]. The properties of melatonin on PE have also been studied in humans. In explants of PE placentas, melatonin did not reduce placental production of activin A, sFlt-1 or sEnd but it reduced oxidative stress, increasing NRF2 and HO-1 expression [157].

## 5. NRF2 Modulation by Synthetic Compounds

Tert-butylhydroquinone (tBHQ) is a synthetic aromatic organic compound, which derives from hydroquinone (substituted with a tert-butyl group). In humans, tBHQ acts as a phase II detoxification enzyme inducer and a NRF2 agonist [158]. In fact, tBHQ increases the level of NRF2, inhibiting its degradation [159]. tBHQ treatment increased NRF2 protein and HO-1 mRNA expression in an HTR-8/SVneo cell line. In addition, these authors found that serum levels of HO-1 protein decreased while placental HO-1 mRNA levels, HO-1 and NRF2 proteins increased in severe PE [160].

Simvastatin is a well-known statin extensively used to reduce the morbidity associated with cardiovascular disease and the risk of developing Alzheimer’s disease. Many studies showed that statins activate HO-1, decrease ROS levels and have antioxidant effects in some cancer cells, fibroblasts and neuronal cells [161]. In trophoblast cells, under experimental hypoxia conditions, simvastatin has been shown to counteract oxidative stress through activation of NRF2 signaling. This activation is dependent on KEAP1 inhibition while NRF2 knockdown resulted in insufficient augmentation of HO-1, GCLC and glutamate–cysteine ligase modifier subunit (GCLM) mRNA expression under oxidative stress [162].

**Figure 2:**
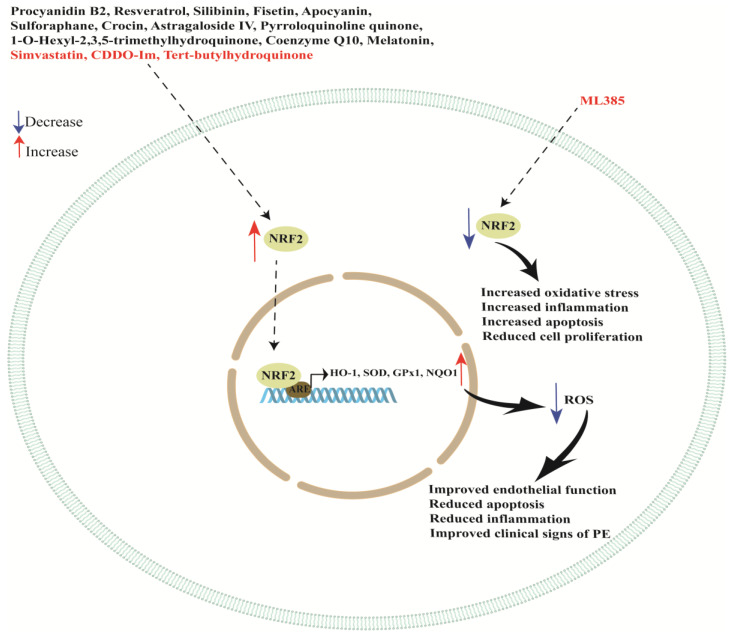
Schematic representation of NRF2 modulation by natural and synthetic compounds. Natural compounds are written in black while synthetic compounds are written in red.

Angiogenesis, inflammation and oxidative stress are among the most important processes involved in PE pathogenesis. An interesting study evaluated the role of NRF2 in modulating these three key processes involved in PE onset. To this aim, Zhang and colleagues found that ML385 (a synthetic NRF2 inhibitor) treatment slightly reduced systolic blood pressure increases and proteinuria in a PE rat model while treatment with CDDO-Im (a NRF2 activator) significantly increased placental HO-1 expression (showing a protective effect) but also increased proteinuria and blood pressure, worsening PE. These authors also found no morphological changes in placentas of rats treated with ML385 and CDDO-Im. However, treatment with ML385 did not lead to significant alteration in HO-1 expression in placental tissues. Moreover, inhibition of NRF2 significantly increased the levels of chemokine 2 (CCL2), interleukin-1β (IL-1β), tumor necrosis factor-alpha (TNF-α), angiotensin II receptor type 1 (AT1R) and ROS in the embryonic tissues. Important results were also obtained in in vitro studies using HTR8/SVneo and hESC cells. In fact, NRF2 knockdown significantly suppressed cell proliferation, improved cell apoptosis and invasion and increased ROS and HO-1 expression. This study showed that inhibition of NRF2 increased oxidative stress, apoptosis and inflammation in the placental tissues but also increased placental angiogenesis and improved fetal and maternal outcomes, suggesting that the relationship between NRF2 and HO-1 expression in PE might not be a simple upstream and downstream relationship but a more complex pathway where other factors play an important role [163]. A schematic representation of NRF2/KEAP1 pathway modulation by natural and synthetic compounds is reported in Figure 2. Studies discussed in this section are summarized in Table 2.

## 6. Conclusions

The NRF2/KEAP1 pathway could be a promising target for treatment of PE, improving or avoiding the onset of this disease. In this review, we discussed several studies focusing on the role of the NRF2/KEAP1 signaling pathway in regulating the oxidative stress response in placental cell lines and in in vivo models. We found that NRF2 can be regulated by important cell modulators such as DJ-1, VEGF, oxLDL, LXA4, AOPPs, miR-133a-3p, CD151 and BRD4 (see Table 1 and Figure 1), favoring the cell response to oxidative stress, avoiding apoptosis and ferroptosis and promoting cell proliferation. Moreover, this pathway plays a key role in important processes for placental development such as hypoxia and syncytiotrophoblast differentiation. These processes are deeply impaired in PE placentas. In addition, we showed that natural and synthetic compounds can act as potent antioxidants, activating the NRF2/KEAP1 signaling pathway, reducing apoptosis and inflammation and improving the clinical signs of PE. In particular, some compounds can improve newborn weight, systolic blood pressure, proteinuria, placental weight and overall fetal survival (see Table 2 and Figure 2).

The studies reviewed above suggest that several compounds and cell modulators can regulate the NRF2/KEAP1 pathway. Thus, these compounds may be used in combination with classical therapy to improve the efficiency of the treatment ameliorating/avoiding PE onset.

## Figures and Tables

**Table 1:** NRF2 cellular modulators.

Modulator	Model Studied	Results	Reference
DJ-1	BeWo cells	NRF2 and GPX4 expression was significantly reduced when DJ-1 was knocked down in BeWo cells. Cell death was significantly increased in DJ-1-deficient cells when these cells were treated with RSL3, a ferroptosis inducer.	[67]
VEGF	BeWo cells	VEGF activated NRF2, increasing thioredoxin (Trx), thioredoxin reductase (TXNRD1) and heme oxygenase-1 (HO-1). VEGF activated NRF2 in an ERK1/2-dependent manner, increasing HO-1 expression then augmenting the production of carbon monoxide, which increased VEGF expression.	[69]
oxLDL	JAR cells and placental explants	Treatment with oxLDL increased NRF2 and HO-1 expression while the blockade of LOX-1 with TS92 inhibited the increase in HO-1 expression induced by oxLDL treatment.	[74]
LXA4	HUVEC	LXA4 inhibited LPS-triggered ROS production, promoting the expression of NRF2 and improving vascular permeability under oxidant stimuli.	[79]
Hypoxia	HTR-8/SVneo cells	NRF2 overexpression in hypoxia-induced cells reduced the levels of MDA and ROS, and decreased ferroptosis.	[83]
Hypoxia	HTR-8/SVneo cells	Hypoxia reduced the activity of CAT, GSH-Px and SOD enzymes and increased NRF2 and HO-1 expression while decreasing KEAP1 expression. The activity of SOD, GSH-Px and CAT in placental tissues of patients with PE was lower compared to normal placental tissues. NRF2 and HO-1 expression in preeclamptic placentas was higher compared to normal pregnancies while KEAP1 expression was lower in PE placentas compared to the normal ones. Silencing NRF2 in HTR8/SVneo cells under hypoxic conditions reduced the activities of CAT, GSH-Px and SOD.	[84]
Hypoxia	PE placentas and HTR-8/SVneo cells	Lower CAT, GSH-Px and SOD activity in HTR8/SVneo cells under hypoxic conditions and in PE placentas. Increased NRF2 and HO-1 expression together with a reduced expression of KEAP1 under hypoxic conditions and in PE placentas.	[85]
AOPPs	HTR-8/SVneo cells	AOPPs increased apoptosis and inhibited the NRF2/ARE/HO-1 pathway. NRF2 silencing aggravated the AOPP-induced cell apoptosis, activating p53 and the caspase cascade while NRF2 overexpression showed cytoprotective effects by increasing HO-1 expression.	[93]
miR-133a-3p	HTR-8/SVneo cells exposed to H_2_O_2_	Transfecting cells with miR-133a-3p under an oxidative stress condition reduced ROS, MDA levels and apoptosis. MiR-133a-3p inhibited BACH1 (a NRF2 repressor), increasing NRF2 activation and HO-1 expression.	[106]
Syncytiotrophoblast differentiation	PE placentas and primary trophoblast cells	NRF2, CYP191A mRNAs and miR-1246 levels were upregulated during syncytiotrophoblast differentiation of trophoblast cells and significantly reduced by hypoxia and in PE placentas. JARID2, Axin-2 and GSK3β expression was significantly downregulated during syncytiotrophoblast differentiation. Silencing of NRF2 in cytotrophoblast cells inhibited miR-1246 and CYP19A1 due to the binding of NRF2 to the miR-1246 and CYP191A promoters.	[111]
CD151	PE placentas, HTR-8/SVneo cells and mice	PE placentas showed reduced expression of CD151, HO-1, NQO1, GCLC and SOD-1. Overexpression of CD151 in HTR-8/SVneo cells enhanced HO-1, NQO1, GCLC and SOD-1 expression. Tail intravenous injection of siCD151 in pregnant mice led to a PE-like phenotype, hypertension and proteinuria. The expression of NRF2, pERK1/2, HO-1, NQO1, GCLC and SOD-1 was decreased in mice and HTR8/SVneo cells when CD151 was silenced. The beneficial effect of CD151 in HTR8/SVneo cells was inhibited when ERK and NRF2 signaling was blocked with synthetic inhibitors.	[114]
BRD4	HTR8/SVneo exposed to H_2_O_2_	BRD4 inhibition attenuated oxidative stress injury by enhancing NRF2 activation via the downregulation of KEAP1.	[116]

**Table 2:** NRF2 modulation by natural and synthetic compounds.

Modulator	Model Studied	Results	Reference
Procyanidin B2	Placental explants and a PE rat model	Procyanidin B2 inhibits sFlt-1 secretion and ameliorates endothelial dysfunction and impaired angiogenesis via the NRF2/PPARγ axis.	[118]
Resveratrol	HUVEC incubated with plasma from PE patients	Antioxidant response element (ARE) activity was increased. The addition of resveratrol by NRF2 activation also occurred.	[120]
Resveratrol	Term placental explants and HUVEC treated with TNF-α and resveratrol	NRF2 knockdown abolished some of the protective effects of resveratrol on endothelial cells, but not in primary trophoblast cells.	[123]
Silibinin	HTR8/SVneo exposed to H_2_O_2_	Silibinin protects the trophoblast from apoptosis, enhancing the activation of NRF2.	[124]
Fisetin	PE rat model	Reduction in hypertension and proteinuria; reduction in TNF-α, IL-6, IL-1β, MDA and the sFlt-1/PlGF ratio; and promoting the NRF2/HO-1 pathway in placental tissues.	[127]
Apocyanin	PE rat model	Reduction in preeclampsia symptoms with combined treatment of apocyanin and aspirin by activating the PI3K/NRF2/HO-1 pathway.	[130]
Sulforaphane	HUVEC and placental explants	In HUVEC, reduction in endothelin-1, VCAM1, ICAM1 and E-selectin. In placental explants, reduction in sFlt-1, endoglin and activin A. In HUVEC, induction of activation and nuclear translocation of NRF2, and induction of HO-1. NRF2 silencing blocked some but not all of sulforaphane’s effects and did not prevent inhibition of trophoblast secretion of sFlt-1 or activin A.	[132]
Crocin	PE rat model	Crocin upregulated protein levels of NRF2 and HO-1.	[134]
Astragaloside IV	PE rat model	Improvements in clinical signs of preeclampsia, reduction in placental cellular oxidative stress and strengthening of the NRF2/HO-1 signaling pathway in placental tissues.	[137]
Pyrroloquinoline quinone	PE rat model	Pyrroloquinoline quinone improved the antioxidation effect in preeclampsia models, activating the NRF2 pathway.	[140]
1-O-hexyl-2,3,5-trimethylhydroquinone (HTHQ)	PE mouse model	HTHQ treatment induced NRF2 expression and nuclear translocation, increasing HO-1 expression in placentas.	[144]
Coenzyme Q10	PE rat model	Coenzyme Q10 protected the rats from preeclampsia through activating the NRF2/HO-1 pathway.	[150]
Melatonin	Pregnant sows	Increase in mRNA levels of antioxidant-related genes involved in the NRF2/ARE pathway (NRF2, SOD, GPx1 and NQO1).	[154]
Melatonin	PE rat model	Increased NRF2, PlGF and HO-1 placental levels with reduction in blood pressure and urine protein content, and recovery in the fetus alive ratio, fetal weight and fetal weight/placental weight ratio.	[155]
Melatonin	Placental explants	Improved oxidative stress, presumably due to the potentiation of NRF2 and HO-1.	[157]
Tert-butylhydroquinone (tBHQ)	HTR-8/SVneo	Increased NRF2 protein and HO-1 mRNA expression after stimulation with tBHQ. HO-1 was located in the cytoplasm and NRF2 was located in both the nucleus and cytoplasm.	[160]
Simvastatin	JAR cells exposed to hypoxia and treated with diethyl maleate (DEM)	In hypoxia conditions, activation of NRF2 signaling depending on KEAP1 inhibition.	[162]
ML385 and CDDO-Im	PE rat model, HTR-8/SVneo and hESC cells	ML385 treatment reduced SBP and proteinuria in PE rats while treatment with CDDO-Im increased proteinuria and systolic blood pressure, worsening PE. HO-1 expression decreased in the PE group compared with the control group while it increased after CDDO-Im treatment compared with the PE group. ML385 did not alter HO-1 expression in placental tissue. NRF2 inhibition increased CCL2, IL-1β, TNF-α, AT1R and ROS in the embryonic tissues. NRF2 knockdown in HTR-8/SVneo and hESC cells suppressed cell proliferation, improved apoptosis and invasion and increased ROS and HO-1 expression.	[163]

## Data Availability

Not applicable.

## References

[1] Costa M.A. (2016). The endocrine function of human placenta: An overview. Reprod. Biomed. Online.

[2] Tossetta G., Avellini C., Licini C., Giannubilo S.R., Castellucci M., Marzioni D. (2016). High temperature requirement A1 and fibronectin: Two possible players in placental tissue remodelling. Eur. J. Histochem..

[3] Chang K.J., Seow K.M., Chen K.H. (2023). Preeclampsia: Recent Advances in Predicting, Preventing, and Managing the Maternal and Fetal Life-Threatening Condition. Int. J. Environ. Res. Public. Health.

[4] Tossetta G., Fantone S., Delli Muti N., Balercia G., Ciavattini A., Giannubilo S.R., Marzioni D. (2022). Preeclampsia and severe acute respiratory syndrome coronavirus 2 infection: A systematic review. J. Hypertens..

[5] Gesuita R., Licini C., Picchiassi E., Tarquini F., Coata G., Fantone S., Tossetta G., Ciavattini A., Castellucci M., Di Renzo G.C. (2019). Association between first trimester plasma htra1 level and subsequent preeclampsia: A possible early marker?. Pregnancy Hypertens..

[6] Burton G.J., Redman C.W., Roberts J.M., Moffett A. (2019). Pre-eclampsia: Pathophysiology and clinical implications. BMJ.

[7] Huppertz B. (2018). The Critical Role of Abnormal Trophoblast Development in the Etiology of Preeclampsia. Curr. Pharm. Biotechnol..

[8] Fantone S., Mazzucchelli R., Giannubilo S.R., Ciavattini A., Marzioni D., Tossetta G. (2020). AT-rich interactive domain 1A protein expression in normal and pathological pregnancies complicated by preeclampsia. Histochem. Cell Biol..

[9] Deshpande J.S., Sundrani D.P., Sahay A.S., Gupte S.A., Joshi S.R. (2021). Unravelling the potential of angiogenic factors for the early prediction of preeclampsia. Hypertens. Res..

[10] Tenorio M.B., Ferreira R.C., Moura F.A., Bueno N.B., de Oliveira A.C.M., Goulart M.O.F. (2019). Cross-Talk between Oxidative Stress and Inflammation in Preeclampsia. Oxidative Med. Cell. Longev..

[11] Cecati M., Sartini D., Campagna R., Biagini A., Ciavattini A., Emanuelli M., Giannubilo S.R. (2017). Molecular analysis of endometrial inflammation in preterm birth. Cell. Mol. Biol..

[12] Licini C., Tossetta G., Avellini C., Ciarmela P., Lorenzi T., Toti P., Gesuita R., Voltolini C., Petraglia F., Castellucci M. (2016). Analysis of cell-cell junctions in human amnion and chorionic plate affected by chorioamnionitis. Histol. Histopathol..

[13] Tossetta G., Fantone S., Gesuita R., Di Renzo G.C., Meyyazhagan A., Tersigni C., Scambia G., Di Simone N., Marzioni D. (2022). HtrA1 in Gestational Diabetes Mellitus: A Possible Biomarker?. Diagnostics.

[14] delli Muti N., Salvio G., Ciarloni A., Perrone M., Tossetta G., Lazzarini R., Bracci M., Balercia G. (2023). Can Extremely Low Frequency Magnetic Field Affect Human Sperm Parameters and Male Fertility?. Tissue Cell.

[15] Didziokaite G., Biliute G., Gudaite J., Kvedariene V. (2023). Oxidative Stress as a Potential Underlying Cause of Minimal and Mild Endometriosis-Related Infertility. Int. J. Mol. Sci..

[16] Shu D.Y., Chaudhary S., Cho K.S., Lennikov A., Miller W.P., Thorn D.C., Yang M., McKay T.B. (2023). Role of Oxidative Stress in Ocular Diseases: A Balancing Act. Metabolites.

[17] Tossetta G., Fantone S., Giannubilo S.R., Marzioni D. (2021). The Multifaced Actions of Curcumin in Pregnancy Outcome. Antioxidants.

[18] Campagna R., Mateuszuk L., Wojnar-Lason K., Kaczara P., Tworzydlo A., Kij A., Bujok R., Mlynarski J., Wang Y., Sartini D. (2021). Nicotinamide N-methyltransferase in endothelium protects against oxidant stress-induced endothelial injury. Biochim. Biophys. Acta Mol. Cell Res..

[19] Zapotoczny B., Braet F., Kus E., Ginda-Makela K., Klejevskaja B., Campagna R., Chlopicki S., Szymonski M. (2019). Actin-spectrin scaffold supports open fenestrae in liver sinusoidal endothelial cells. Traffic.

[20] Iantomasi T., Romagnoli C., Palmini G., Donati S., Falsetti I., Miglietta F., Aurilia C., Marini F., Giusti F., Brandi M.L. (2023). Oxidative Stress and Inflammation in Osteoporosis: Molecular Mechanisms Involved and the Relationship with microRNAs. Int. J. Mol. Sci..

[21] Moratilla-Rivera I., Sanchez M., Valdes-Gonzalez J.A., Gomez-Serranillos M.P. (2023). Natural Products as Modulators of Nrf2 Signaling Pathway in Neuroprotection. Int. J. Mol. Sci..

[22] Tossetta G., Marzioni D. (2022). Natural and synthetic compounds in Ovarian Cancer: A focus on NRF2/KEAP1 pathway. Pharmacol. Res..

[23] Marzioni D., Mazzucchelli R., Fantone S., Tossetta G. (2023). NRF2 modulation in TRAMP mice: An in vivo model of prostate cancer. Mol. Biol. Rep..

[24] Tossetta G., Marzioni D. (2023). Targeting the NRF2/KEAP1 pathway in cervical and endometrial cancers. Eur. J. Pharmacol..

[25] Tossetta G., Fantone S., Montanari E., Marzioni D., Goteri G. (2022). Role of NRF2 in Ovarian Cancer. Antioxidants.

[26] Emanuelli M., Sartini D., Molinelli E., Campagna R., Pozzi V., Salvolini E., Simonetti O., Campanati A., Offidani A. (2022). The Double-Edged Sword of Oxidative Stress in Skin Damage and Melanoma: From Physiopathology to Therapeutical Approaches. Antioxidants.

[27] Sartini D., Campagna R., Lucarini G., Pompei V., Salvolini E., Mattioli-Belmonte M., Molinelli E., Brisigotti V., Campanati A., Bacchetti T. (2021). Differential immunohistochemical expression of paraoxonase-2 in actinic keratosis and squamous cell carcinoma. Hum. Cell.

[28] Torres-Cuevas I., Parra-Llorca A., Sanchez-Illana A., Nunez-Ramiro A., Kuligowski J., Chafer-Pericas C., Cernada M., Escobar J., Vento M. (2017). Oxygen and oxidative stress in the perinatal period. Redox Biol..

[29] Taysi S., Tascan A.S., Ugur M.G., Demir M. (2019). Radicals, Oxidative/Nitrosative Stress and Preeclampsia. Mini Rev. Med. Chem..

[30] Teimoori B., Moradi-Shahrebabak M., Razavi M., Rezaei M., Harati-Sadegh M., Salimi S. (2019). The effect of GPx-1 rs1050450 and MnSOD rs4880 polymorphisms on PE susceptibility: A case-control study. Mol. Biol. Rep..

[31] Luo Z.C., Julien P., Wei S.Q., Audibert F., Fraser W.D., Maternal and Infant Research on Oxidative Stress study group (2018). Association of pre-eclampsia with SOD2 Ala16Val polymorphism among mother-father-infant triads. Int. J. Gynaecol. Obstet..

[32] Szczesny-Malysiak E., Stojak M., Campagna R., Grosicki M., Jamrozik M., Kaczara P., Chlopicki S. (2020). Bardoxolone Methyl Displays Detrimental Effects on Endothelial Bioenergetics, Suppresses Endothelial ET-1 Release, and Increases Endothelial Permeability in Human Microvascular Endothelium. Oxidative Med. Cell. Longev..

[33] Baird L., Yamamoto M. (2020). The Molecular Mechanisms Regulating the KEAP1-NRF2 Pathway. Mol. Cell. Biol..

[34] Ahmed S.M., Luo L., Namani A., Wang X.J., Tang X. (2017). Nrf2 signaling pathway: Pivotal roles in inflammation. Biochim. Biophys. Acta Mol. Basis Dis..

[35] Shahcheraghi S.H., Salemi F., Small S., Syed S., Salari F., Alam W., Cheang W.S., Saso L., Khan H. (2023). Resveratrol regulates inflammation and improves oxidative stress via Nrf2 signaling pathway: Therapeutic and biotechnological prospects. Phytother. Res..

[36] Catanzaro E., Calcabrini C., Turrini E., Sestili P., Fimognari C. (2017). Nrf2: A potential therapeutic target for naturally occurring anticancer drugs?. Expert. Opin. Ther. Targets.

[37] Hamzawy M.A., Abo-Youssef A.M., Malak M.N., Khalaf M.M. (2022). Multiple targets of Nrf 2 inhibitor; trigonelline in combating urethane-induced lung cancer by caspase-executioner apoptosis, cGMP and limitation of cyclin D1 and Bcl2. Eur. Rev. Med. Pharmacol. Sci..

[38] Ni C., Ye Q., Mi X., Jiao D., Zhang S., Cheng R., Fang Z., Fang M., Ye X. (2023). Resveratrol inhibits ferroptosis via activating NRF2/GPX4 pathway in mice with spinal cord injury. Microsc. Res. Tech..

[39] Martin K.R., Barrett J.C. (2002). Reactive oxygen species as double-edged swords in cellular processes: Low-dose cell signaling versus high-dose toxicity. Hum. Exp. Toxicol..

[40] Sauer H., Wartenberg M., Hescheler J. (2001). Reactive oxygen species as intracellular messengers during cell growth and differentiation. Cell Physiol. Biochem..

[41] Fantone S., Tossetta G., Di Simone N., Tersigni C., Scambia G., Marcheggiani F., Giannubilo S.R., Marzioni D. (2022). CD93 a potential player in cytotrophoblast and endothelial cell migration. Cell Tissue Res..

[42] Margioula-Siarkou G., Margioula-Siarkou C., Petousis S., Margaritis K., Vavoulidis E., Gullo G., Alexandratou M., Dinas K., Sotiriadis A., Mavromatidis G. (2022). The role of endoglin and its soluble form in pathogenesis of preeclampsia. Mol. Cell. Biochem..

[43] Kweider N., Huppertz B., Kadyrov M., Rath W., Pufe T., Wruck C.J. (2014). A possible protective role of Nrf2 in preeclampsia. Ann. Anat..

[44] Smith S.C., Guilbert L.J., Yui J., Baker P.N., Davidge S.T. (1999). The role of reactive nitrogen/oxygen intermediates in cytokine-induced trophoblast apoptosis. Placenta.

[45] Mam V., Tanbe A.F., Vitali S.H., Arons E., Christou H.A., Khalil R.A. (2010). Impaired vasoconstriction and nitric oxide-mediated relaxation in pulmonary arteries of hypoxia- and monocrotaline-induced pulmonary hypertensive rats. J. Pharmacol. Exp. Ther..

[46] Mansego M.L., Solar Gde M., Alonso M.P., Martinez F., Saez G.T., Escudero J.C., Redon J., Chaves F.J. (2011). Polymorphisms of antioxidant enzymes, blood pressure and risk of hypertension. J. Hypertens..

[47] George E.M., Granger J.P. (2013). Heme oxygenase in pregnancy and preeclampsia. Curr. Opin. Nephrol. Hypertens..

[48] Li Y., Yu X.J., Xiao T., Chi H.L., Zhu G.Q., Kang Y.M. (2021). Nrf1 Knock-Down in the Hypothalamic Paraventricular Nucleus Alleviates Hypertension Through Intervention of Superoxide Production-Removal Balance and Mitochondrial Function. Cardiovasc. Toxicol..

[49] Gomes P., Simao S., Lemos V., Amaral J.S., Soares-da-Silva P. (2013). Loss of oxidative stress tolerance in hypertension is linked to reduced catalase activity and increased c-Jun NH2-terminal kinase activation. Free Radic. Biol. Med..

[50] Li J., Zhang C., Xing Y., Janicki J.S., Yamamoto M., Wang X.L., Tang D.Q., Cui T. (2011). Up-regulation of p27(kip1) contributes to Nrf2-mediated protection against angiotensin II-induced cardiac hypertrophy. Cardiovasc. Res..

[51] Nezu M., Souma T., Yu L., Sekine H., Takahashi N., Wei A.Z., Ito S., Fukamizu A., Zsengeller Z.K., Nakamura T. (2017). Nrf2 inactivation enhances placental angiogenesis in a preeclampsia mouse model and improves maternal and fetal outcomes. Sci. Signal..

[52] Giretti I., D’Ascenzo R., Correani A., Antognoli L., Monachesi C., Biagetti C., Pompilio A., Marinelli L., Burattini I., Cogo P. (2021). Hypertriglyceridemia and lipid tolerance in preterm infants with a birth weight of less than 1250 g on routine parenteral nutrition. Clin. Nutr..

[53] Nobile S., Marchionni P., Gidiucci C., Correani A., Palazzi M.L., Spagnoli C., Rondina C., Marche Neonatal N., Carnielli V.P. (2019). Oxygen saturation/FIO2 ratio at 36 weeks’ PMA in 1005 preterm infants: Effect of gestational age and early respiratory disease patterns. Pediatr. Pulmonol..

[54] Mundal S.B., Rakner J.J., Silva G.B., Gierman L.M., Austdal M., Basnet P., Elschot M., Bakke S.S., Ostrop J., Thomsen L.C.V. (2022). Divergent Regulation of Decidual Oxidative-Stress Response by NRF2 and KEAP1 in Preeclampsia with and without Fetal Growth Restriction. Int. J. Mol. Sci..

[55] Loset M., Mundal S.B., Johnson M.P., Fenstad M.H., Freed K.A., Lian I.A., Eide I.P., Bjorge L., Blangero J., Moses E.K. (2011). A transcriptional profile of the decidua in preeclampsia. Am. J. Obstet. Gynecol..

[56] Kweider N., Huppertz B., Wruck C.J., Beckmann R., Rath W., Pufe T., Kadyrov M. (2012). A role for Nrf2 in redox signalling of the invasive extravillous trophoblast in severe early onset IUGR associated with preeclampsia. PLoS ONE.

[57] Acar N., Soylu H., Edizer I., Ozbey O., Er H., Akkoyunlu G., Gemici B., Ustunel I. (2014). Expression of nuclear factor erythroid 2-related factor 2 (Nrf2) and peroxiredoxin 6 (Prdx6) proteins in healthy and pathologic placentas of human and rat. Acta Histochem..

[58] Wruck C.J., Huppertz B., Bose P., Brandenburg L.O., Pufe T., Kadyrov M. (2009). Role of a fetal defence mechanism against oxidative stress in the aetiology of preeclampsia. Histopathology.

[59] Correani A., Visentin S., Cosmi E., Ponchia E., D’Aronco S., Simonato M., Vedovelli L., Cogo P., Carnielli V.P. (2018). The maternal-fetal gradient of free and esterified phytosterols at the time of delivery in humans. Clin. Nutr..

[60] Borst P., Evers R., Kool M., Wijnholds J. (2000). A family of drug transporters: The multidrug resistance-associated proteins. J. Natl. Cancer Inst..

[61] Ji L., Li H., Gao P., Shang G., Zhang D.D., Zhang N., Jiang T. (2013). Nrf2 pathway regulates multidrug-resistance-associated protein 1 in small cell lung cancer. PLoS ONE.

[62] Yu L., Wang T., Que R., Yang J., Wang Z., Jiang X., Wang L. (2019). The potentially protective role of ATP-binding cassette transporters in preeclampsia via Nrf2. Pregnancy Hypertens..

[63] Ma H., Dong Y., Chu Y., Guo Y., Li L. (2022). The mechanisms of ferroptosis and its role in alzheimer’s disease. Front. Mol. Biosci..

[64] Morris G., Berk M., Carvalho A.F., Maes M., Walker A.J., Puri B.K. (2018). Why should neuroscientists worry about iron? The emerging role of ferroptosis in the pathophysiology of neuroprogressive diseases. Behav. Brain Res..

[65] Cao J., Chen X., Jiang L., Lu B., Yuan M., Zhu D., Zhu H., He Q., Yang B., Ying M. (2020). DJ-1 suppresses ferroptosis through preserving the activity of S-adenosyl homocysteine hydrolase. Nat. Commun..

[66] Kwon H.S., Hwang H.S., Sohn I.S., Park S.H. (2013). Expression of DJ-1 proteins in placentas from women with severe preeclampsia. Eur. J. Obstet. Gynecol. Reprod. Biol..

[67] Liao T., Xu X., Ye X., Yan J. (2022). DJ-1 upregulates the Nrf2/GPX4 signal pathway to inhibit trophoblast ferroptosis in the pathogenesis of preeclampsia. Sci. Rep..

[68] Martinez-Casales M., Hernanz R., Alonso M.J. (2021). Vascular and Macrophage Heme Oxygenase-1 in Hypertension: A Mini-Review. Front. Physiol..

[69] Kweider N., Fragoulis A., Rosen C., Pecks U., Rath W., Pufe T., Wruck C.J. (2011). Interplay between vascular endothelial growth factor (VEGF) and nuclear factor erythroid 2-related factor-2 (Nrf2): Implications for preeclampsia. J. Biol. Chem..

[70] Branch D.W., Mitchell M.D., Miller E., Palinski W., Witztum J.L. (1994). Pre-eclampsia and serum antibodies to oxidised low-density lipoprotein. Lancet.

[71] Uzun H., Benian A., Madazli R., Topcuoglu M.A., Aydin S., Albayrak M. (2005). Circulating oxidized low-density lipoprotein and paraoxonase activity in preeclampsia. Gynecol. Obstet. Investig..

[72] Tsumita T., Maishi N., Annan D.A., Towfik M.A., Matsuda A., Onodera Y., Nam J.M., Hida Y., Hida K. (2022). The oxidized-LDL/LOX-1 axis in tumor endothelial cells enhances metastasis by recruiting neutrophils and cancer cells. Int. J. Cancer.

[73] Ishii T., Itoh K., Ruiz E., Leake D.S., Unoki H., Yamamoto M., Mann G.E. (2004). Role of Nrf2 in the regulation of CD36 and stress protein expression in murine macrophages: Activation by oxidatively modified LDL and 4-hydroxynonenal. Circ. Res..

[74] Chigusa Y., Tatsumi K., Kondoh E., Fujita K., Nishimura F., Mogami H., Konishi I. (2012). Decreased lectin-like oxidized LDL receptor 1 (LOX-1) and low Nrf2 activation in placenta are involved in preeclampsia. J. Clin. Endocrinol. Metab..

[75] Gupta S., Agarwal A., Sharma R.K. (2005). The role of placental oxidative stress and lipid peroxidation in preeclampsia. Obstet. Gynecol. Surv..

[76] Serhan C.N., Chiang N., Van Dyke T.E. (2008). Resolving inflammation: Dual anti-inflammatory and pro-resolution lipid mediators. Nat. Rev. Immunol..

[77] Xu Z., Zhao F., Lin F., Xiang H., Wang N., Ye D., Huang Y. (2014). Preeclampsia is associated with a deficiency of lipoxin A4, an endogenous anti-inflammatory mediator. Fertil. Steril..

[78] Lin F., Zeng P., Xu Z., Ye D., Yu X., Wang N., Tang J., Zhou Y., Huang Y. (2012). Treatment of Lipoxin A(4) and its analogue on low-dose endotoxin induced preeclampsia in rat and possible mechanisms. Reprod. Toxicol..

[79] Pang H., Yi P., Wu P., Liu Z., Liu Z., Gong J., Hao H., Cai L., Ye D., Huang Y. (2011). Effect of lipoxin A4 on lipopolysaccharide-induced endothelial hyperpermeability. Sci. World J..

[80] Hu X.Q., Zhang L. (2021). Hypoxia and the integrated stress response promote pulmonary hypertension and preeclampsia: Implications in drug development. Drug Discov. Today.

[81] Goldman-Wohl D., Yagel S. (2002). Regulation of trophoblast invasion: From normal implantation to pre-eclampsia. Mol. Cell. Endocrinol..

[82] Xie Y., Hou W., Song X., Yu Y., Huang J., Sun X., Kang R., Tang D. (2016). Ferroptosis: Process and function. Cell. Death Differ..

[83] Wang Y., Zhang L., Zhou X. (2021). Activation of Nrf2 signaling protects hypoxia-induced HTR-8/SVneo cells against ferroptosis. J. Obstet. Gynaecol. Res..

[84] Qiu D., Wu J., Li M., Wang L., Zhu X., Chen Y. (2021). Impaction of factors associated with oxidative stress on the pathogenesis of gestational hypertension and preeclampsia: A Chinese patients based study. Medicine.

[85] Feng H., Wang L., Zhang G., Zhang Z., Guo W. (2020). Oxidative stress activated by Keap-1/Nrf2 signaling pathway in pathogenesis of preeclampsia. Int. J. Clin. Exp. Pathol..

[86] Bayarsaikhan G., Bayarsaikhan D., Lee J., Lee B. (2022). Targeting Scavenger Receptors in Inflammatory Disorders and Oxidative Stress. Antioxidants.

[87] Kalousova M., Zima T., Tesar V., Dusilova-Sulkova S., Skrha J. (2005). Advanced glycoxidation end products in chronic diseases-clinical chemistry and genetic background. Mutat. Res..

[88] Ungurianu A., Zanfirescu A., Gradinaru D., Ionescu-Tirgoviste C., Danciulescu Miulescu R., Margina D. (2022). Interleukins and redox impairment in type 2 diabetes mellitus: Mini-review and pilot study. Curr. Med. Res. Opin..

[89] Stanek A., Brozyna-Tkaczyk K., Myslinski W. (2021). Oxidative Stress Markers among Obstructive Sleep Apnea Patients. Oxidative Med. Cell. Longev..

[90] Zhao Y., Zhang L., Ouyang X., Jiang Z., Xie Z., Fan L., Zhu D., Li L. (2019). Advanced oxidation protein products play critical roles in liver diseases. Eur. J. Clin. Investig..

[91] Cristani M., Speciale A., Saija A., Gangemi S., Minciullo P.L., Cimino F. (2016). Circulating Advanced Oxidation Protein Products as Oxidative Stress Biomarkers and Progression Mediators in Pathological Conditions Related to Inflammation and Immune Dysregulation. Curr. Med. Chem..

[92] Wang S.S., Huang Q.T., Zhong M., Yin Q. (2015). AOPPs (advanced oxidation protein products) promote apoptosis in trophoblastic cells through interference with NADPH oxidase signaling: Implications for preeclampsia. J. Matern.-Fetal Neonatal Med..

[93] Chen S., Yin Q., Hu H., Chen Q., Huang Q., Zhong M. (2021). AOPPs induce HTR-8/SVneo cell apoptosis by downregulating the Nrf-2/ARE/HO-1 anti-oxidative pathway: Potential implications for preeclampsia. Placenta.

[94] Avellini C., Licini C., Lazzarini R., Gesuita R., Guerra E., Tossetta G., Castellucci C., Giannubilo S.R., Procopio A., Alberti S. (2017). The trophoblast cell surface antigen 2 and miR-125b axis in urothelial bladder cancer. Oncotarget.

[95] Karimi Dermani F., Datta I., Gholamzadeh Khoei S. (2023). MicroRNA-452: A double-edged sword in multiple human cancers. Clin. Transl. Oncol..

[96] Li L., Xun C., Yu C.H. (2022). Role of microRNA-regulated cancer stem cells in recurrent hepatocellular carcinoma. World J. Hepatol..

[97] Poniewierska-Baran A., Sluczanowska-Glabowska S., Malkowska P., Sierawska O., Zadroga L., Pawlik A., Niedzwiedzka-Rystwej P. (2022). Role of miRNA in Melanoma Development and Progression. Int. J. Mol. Sci..

[98] Licini C., Avellini C., Picchiassi E., Mensa E., Fantone S., Ramini D., Tersigni C., Tossetta G., Castellucci C., Tarquini F. (2021). Pre-eclampsia predictive ability of maternal miR-125b: A clinical and experimental study. Transl. Res..

[99] Atic A.I., Thiele M., Munk A., Dalgaard L.T. (2023). Circulating microRNAs associated with non-alcoholic fatty liver disease. Am. J. Physiol. Cell Physiol..

[100] Elkhawaga S.Y., Ismail A., Elsakka E.G.E., Doghish A.S., Elkady M.A., El-Mahdy H.A. (2023). miRNAs as cornerstones in adipogenesis and obesity. Life Sci..

[101] Tofigh R., Hosseinpourfeizi M., Baradaran B., Teimourian S., Safaralizadeh R. (2023). Rheumatoid arthritis and non-coding RNAs; how to trigger inflammation. Life Sci..

[102] Nohata N., Hanazawa T., Enokida H., Seki N. (2012). microRNA-1/133a and microRNA-206/133b clusters: Dysregulation and functional roles in human cancers. Oncotarget.

[103] Yu H., Lu Y., Li Z., Wang Q. (2014). microRNA-133: Expression, function and therapeutic potential in muscle diseases and cancer. Curr. Drug Targets.

[104] Xiao Y., Zhao J., Tuazon J.P., Borlongan C.V., Yu G. (2019). MicroRNA-133a and Myocardial Infarction. Cell. Transplant..

[105] Mafra D., Alvarenga L., Cardozo L., Stockler-Pinto M.B., Nakao L.S., Stenvinkel P., Shiels P.G. (2022). Inhibiting BTB domain and CNC homolog 1 (Bach1) as an alternative to increase Nrf2 activation in chronic diseases. Biochim. Biophys. Acta Gen. Subj..

[106] Guo H., Wang Y., Jia W., Liu L. (2021). MiR-133a-3p relieves the oxidative stress induced trophoblast cell apoptosis through the BACH1/Nrf2/HO-1 signaling pathway. Physiol. Res..

[107] Takeshita N., Hoshino I., Mori M., Akutsu Y., Hanari N., Yoneyama Y., Ikeda N., Isozaki Y., Maruyama T., Akanuma N. (2013). Serum microRNA expression profile: miR-1246 as a novel diagnostic and prognostic biomarker for oesophageal squamous cell carcinoma. Br. J. Cancer.

[108] Zhang Z., Wang X., Zhang L., Shi Y., Wang J., Yan H. (2017). Wnt/beta-catenin signaling pathway in trophoblasts and abnormal activation in preeclampsia (Review). Mol. Med. Rep..

[109] Fournet-Dulguerov N., MacLusky N.J., Leranth C.Z., Todd R., Mendelson C.R., Simpson E.R., Naftolin F. (1987). Immunohistochemical localization of aromatase cytochrome P-450 and estradiol dehydrogenase in the syncytiotrophoblast of the human placenta. J. Clin. Endocrinol. Metab..

[110] Shen X., Kim W., Fujiwara Y., Simon M.D., Liu Y., Mysliwiec M.R., Yuan G.C., Lee Y., Orkin S.H. (2009). Jumonji modulates polycomb activity and self-renewal versus differentiation of stem cells. Cell.

[111] Muralimanoharan S., Kwak Y.T., Mendelson C.R. (2018). Redox-Sensitive Transcription Factor NRF2 Enhances Trophoblast Differentiation via Induction of miR-1246 and Aromatase. Endocrinology.

[112] Lin W., Liu J., Chen J., Li J., Qiu S., Ma J., Lin X., Zhang L., Wu J. (2019). Peptides of tetraspanin oncoprotein CD151 trigger active immunity against primary tumour and experimental lung metastasis. eBioMedicine.

[113] Randhawa P.K., Rajakumar A., Futuro de Lima I.B., Gupta M.K. (2023). Eugenol attenuates ischemia-mediated oxidative stress in cardiomyocytes via acetylation of histone at H3K27. Free Radic. Biol. Med..

[114] Wang Z., Cai B., Cao C., Lv H., Dai Y., Zheng M., Zhao G., Peng Y., Gou W., Wang J. (2021). Downregulation of CD151 induces oxidative stress and apoptosis in trophoblast cells via inhibiting ERK/Nrf2 signaling pathway in preeclampsia. Free Radic. Biol. Med..

[115] Hussong M., Borno S.T., Kerick M., Wunderlich A., Franz A., Sultmann H., Timmermann B., Lehrach H., Hirsch-Kauffmann M., Schweiger M.R. (2014). The bromodomain protein BRD4 regulates the KEAP1/NRF2-dependent oxidative stress response. Cell Death Dis..

[116] Wu Y., Mi Y., Zhang F., Cheng Y., Wu X. (2021). Suppression of bromodomain-containing protein 4 protects trophoblast cells from oxidative stress injury by enhancing Nrf2 activation. Hum. Exp. Toxicol..

[117] Nie X., Tang W., Zhang Z., Yang C., Qian L., Xie X., Qiang E., Zhao J., Zhao W., Xiao L. (2020). Procyanidin B2 mitigates endothelial endoplasmic reticulum stress through a PPARdelta-Dependent mechanism. Redox Biol..

[118] Liu L., Wang R., Xu R., Chu Y., Gu W. (2022). Procyanidin B2 ameliorates endothelial dysfunction and impaired angiogenesis via the Nrf2/PPARgamma/sFlt-1 axis in preeclampsia. Pharmacol. Res..

[119] Ungvari Z., Bagi Z., Feher A., Recchia F.A., Sonntag W.E., Pearson K., de Cabo R., Csiszar A. (2010). Resveratrol confers endothelial protection via activation of the antioxidant transcription factor Nrf2. Am. J. Physiol. Heart Circ. Physiol..

[120] Caldeira-Dias M., Viana-Mattioli S., de Souza Rangel Machado J., Carlstrom M., de Carvalho Cavalli R., Sandrim V.C. (2021). Resveratrol and grape juice: Effects on redox status and nitric oxide production of endothelial cells in in vitro preeclampsia model. Pregnancy Hypertens..

[121] George E.M., Colson D., Dixon J., Palei A.C., Granger J.P. (2012). Heme Oxygenase-1 Attenuates Hypoxia-Induced sFlt-1 and Oxidative Stress in Placental Villi through Its Metabolic Products CO and Bilirubin. Int. J. Hypertens..

[122] Lim R., Acharya R., Delpachitra P., Hobson S., Sobey C.G., Drummond G.R., Wallace E.M. (2015). Activin and NADPH-oxidase in preeclampsia: Insights from in vitro and murine studies. Am. J. Obstet. Gynecol..

[123] Gurusinghe S., Cox A.G., Rahman R., Chan S.T., Muljadi R., Singh H., Leaw B., Mockler J.C., Marshall S.A., Murthi P. (2017). Resveratrol mitigates trophoblast and endothelial dysfunction partly via activation of nuclear factor erythroid 2-related factor-2. Placenta.

[124] Guo H., Wang Y., Liu D. (2020). Silibinin ameliorats H_2_O_2_-induced cell apoptosis and oxidative stress response by activating Nrf2 signaling in trophoblast cells. Acta Histochem..

[125] Kim S., Choi K.J., Cho S.J., Yun S.M., Jeon J.P., Koh Y.H., Song J., Johnson G.V., Jo C. (2016). Fisetin stimulates autophagic degradation of phosphorylated tau via the activation of TFEB and Nrf2 transcription factors. Sci. Rep..

[126] Ehren J.L., Maher P. (2013). Concurrent regulation of the transcription factors Nrf2 and ATF4 mediates the enhancement of glutathione levels by the flavonoid fisetin. Biochem. Pharmacol..

[127] Li Y., Liu Y., Chen J., Hu J. (2022). Protective effect of Fisetin on the lipopolysaccharide-induced preeclampsia-like rats. Hypertens. Pregnancy.

[128] Savla S.R., Laddha A.P., Kulkarni Y.A. (2021). Pharmacology of apocynin: A natural acetophenone. Drug. Metab. Rev..

[129] Barbieri S.S., Cavalca V., Eligini S., Brambilla M., Caiani A., Tremoli E., Colli S. (2004). Apocynin prevents cyclooxygenase 2 expression in human monocytes through NADPH oxidase and glutathione redox-dependent mechanisms. Free Radic. Biol. Med..

[130] Ju Y., Feng Y., Hou X., Wu L., Yang H., Zhang H., Ma Y. (2022). Combined apocyanin and aspirin treatment activates the PI3K/Nrf2/HO-1 signaling pathway and ameliorates preeclampsia symptoms in rats. Hypertens. Pregnancy.

[131] Mizuno K., Kume T., Muto C., Takada-Takatori Y., Izumi Y., Sugimoto H., Akaike A. (2011). Glutathione biosynthesis via activation of the nuclear factor E2-related factor 2 (Nrf2)—Antioxidant-response element (ARE) pathway is essential for neuroprotective effects of sulforaphane and 6-(methylsulfinyl) hexyl isothiocyanate. J. Pharmacol. Sci..

[132] Cox A.G., Gurusinghe S., Abd Rahman R., Leaw B., Chan S.T., Mockler J.C., Murthi P., Marshall S.A., Lim R., Wallace E.M. (2019). Sulforaphane improves endothelial function and reduces placental oxidative stress in vitro. Pregnancy Hypertens..

[133] Mashmoul M., Azlan A., Mohtarrudin N., Yusof B.N.M., Khaza’ai H. (2017). Saffron extract and crocin reduced biomarkers associated with obesity in rats fed a high-fat diet. Malays. J. Nutr..

[134] Chen X., Huang J., Lv Y., Chen Y., Rao J. (2021). Crocin exhibits an antihypertensive effect in a rat model of gestational hypertension and activates the Nrf-2/HO-1 signaling pathway. Hypertens. Res..

[135] Qian W., Cai X., Qian Q., Zhuang Q., Yang W., Zhang X., Zhao L. (2019). Astragaloside IV protects endothelial progenitor cells from the damage of ox-LDL via the LOX-1/NLRP3 inflammasome pathway. Drug Des. Devel Ther..

[136] Liu Y.L., Zhang Q.Z., Wang Y.R., Fu L.N., Han J.S., Zhang J., Wang B.M. (2020). Astragaloside IV Improves High-Fat Diet-Induced Hepatic Steatosis in Nonalcoholic Fatty Liver Disease Rats by Regulating Inflammatory Factors Level via TLR4/NF-kappaB Signaling Pathway. Front. Pharmacol..

[137] Yang S., Zhang R., Xing B., Zhou L., Zhang P., Song L. (2020). Astragaloside IV ameliorates preeclampsia-induced oxidative stress through the Nrf2/HO-1 pathway in a rat model. Am. J. Physiol. Endocrinol. Metab..

[138] Ikemoto K., Mori S., Mukai K. (2017). Synthesis and crystal structure of pyrroloquinoline quinol (PQQH(2)) and pyrroloquinoline quinone (PQQ). Acta Crystallogr. B Struct. Sci. Cryst. Eng. Mater..

[139] Hwang P., Willoughby D.S. (2018). Mechanisms Behind Pyrroloquinoline Quinone Supplementation on Skeletal Muscle Mitochondrial Biogenesis: Possible Synergistic Effects with Exercise. J. Am. Coll. Nutr..

[140] Wang H., Li M., Chen P., Shi X. (2022). Anti-inflammatory and Antioxidant Effects of Pyrroloquinoline Quinone in L-NAME-Induced Preeclampsia-Like Rat Model. Reprod. Sci..

[141] Kim J., Shin S.H., Kang J.K., Kim J.W. (2018). HX-1171 attenuates pancreatic beta-cell apoptosis and hyperglycemia-mediated oxidative stress via Nrf2 activation in streptozotocin-induced diabetic model. Oncotarget.

[142] Yada H., Hirose M., Tamano S., Kawabe M., Sano M., Takahashi S., Futakuchi M., Miki T., Shirai T. (2002). Effects of antioxidant 1-O-hexyl-2,3,5-trimethylhydroquinone or ascorbic acid on carcinogenesis induced by administration of aminopyrine and sodium nitrite in a rat multi-organ carcinogenesis model. Jpn. J. Cancer Res..

[143] Park H.J., Kang J.K., Lee M.K. (2019). 1-O-Hexyl-2,3,5-Trimethylhydroquinone Ameliorates l-DOPA-Induced Cytotoxicity in PC12 Cells. Molecules.

[144] Jiang L., Gong Y., Rao J., Yang Q., Gao N., Li G., Ma Y. (2021). 1-O-Hexyl-2,3,5-Trimethylhydroquinone Ameliorates the Development of Preeclampsia through Suppression of Oxidative Stress and Endothelial Cell Apoptosis. Oxidative Med. Cell. Longev..

[145] Hornos Carneiro M.F., Colaiacovo M.P. (2023). Beneficial antioxidant effects of Coenzyme Q10 on reproduction. Vitam. Horm..

[146] Mantle D., Lopez-Lluch G., Hargreaves I.P. (2023). Coenzyme Q10 Metabolism: A Review of Unresolved Issues. Int. J. Mol. Sci..

[147] Witting P.K., Pettersson K., Letters J., Stocker R. (2000). Anti-atherogenic effect of coenzyme Q10 in apolipoprotein E gene knockout mice. Free Radic. Biol. Med..

[148] Singh R.B., Shinde S.N., Chopra R.K., Niaz M.A., Thakur A.S., Onouchi Z. (2000). Effect of coenzyme Q10 on experimental atherosclerosis and chemical composition and quality of atheroma in rabbits. Atherosclerosis.

[149] Palan P.R., Shaban D.W., Martino T., Mikhail M.S. (2004). Lipid-soluble antioxidants and pregnancy: Maternal serum levels of coenzyme Q10, alpha-tocopherol and gamma-tocopherol in preeclampsia and normal pregnancy. Gynecol. Obstet. Investig..

[150] Li L., Li H., Zhou Q., Lu Y., Chen P., Wang X., Zhao H. (2020). Implication of nuclear factor-erythroid 2-like 2/heme oxygenase 1 pathway in the protective effects of coenzyme Q10 against preeclampsia-like in a rat model. Microcirculation.

[151] Lanoix D., Beghdadi H., Lafond J., Vaillancourt C. (2008). Human placental trophoblasts synthesize melatonin and express its receptors. J. Pineal Res..

[152] Socaciu A.I., Ionut R., Socaciu M.A., Ungur A.P., Barsan M., Chiorean A., Socaciu C., Rajnoveanu A.G. (2020). Melatonin, an ubiquitous metabolic regulator: Functions, mechanisms and effects on circadian disruption and degenerative diseases. Rev. Endocr. Metab. Disord..

[153] Zeng K., Gao Y., Wan J., Tong M., Lee A.C., Zhao M., Chen Q. (2016). The reduction in circulating levels of melatonin may be associated with the development of preeclampsia. J. Hum. Hypertens..

[154] Peng X., Cai X., Li J., Huang Y., Liu H., He J., Fang Z., Feng B., Tang J., Lin Y. (2021). Effects of Melatonin Supplementation during Pregnancy on Reproductive Performance, Maternal-Placental-Fetal Redox Status, and Placental Mitochondrial Function in a Sow Model. Antioxidants.

[155] Zuo J., Jiang Z. (2020). Melatonin attenuates hypertension and oxidative stress in a rat model of L-NAME-induced gestational hypertension. Vasc. Med..

[156] Henderson J.T., O’Connor E., Whitlock E.P. (2014). Low-dose aspirin for prevention of morbidity and mortality from preeclampsia. Ann. Intern. Med..

[157] Hobson S.R., Gurusinghe S., Lim R., Alers N.O., Miller S.L., Kingdom J.C., Wallace E.M. (2018). Melatonin improves endothelial function in vitro and prolongs pregnancy in women with early-onset preeclampsia. J. Pineal Res..

[158] Ye F., Li X., Li L., Yuan J., Chen J. (2016). t-BHQ Provides Protection against Lead Neurotoxicity via Nrf2/HO-1 Pathway. Oxidative Med. Cell. Longev..

[159] Zhao R., Yang B., Wang L., Xue P., Deng B., Zhang G., Jiang S., Zhang M., Liu M., Pi J. (2013). Curcumin protects human keratinocytes against inorganic arsenite-induced acute cytotoxicity through an NRF2-dependent mechanism. Oxidative Med. Cell. Longev..

[160] Li J., Zhou J., Ye Y., Liu Q., Wang X., Zhang N., Wang X. (2016). Increased Heme Oxygenase-1 and Nuclear Factor Erythroid 2-Related Factor-2 in the Placenta Have a Cooperative Action on Preeclampsia. Gynecol. Obstet. Investig..

[161] Chartoumpekis D., Ziros P.G., Psyrogiannis A., Kyriazopoulou V., Papavassiliou A.G., Habeos I.G. (2010). Simvastatin lowers reactive oxygen species level by Nrf2 activation via PI3K/Akt pathway. Biochem. Biophys. Res. Commun..

[162] Chigusa Y., Kawasaki K., Kondoh E., Mogami H., Ujita M., Fujita K., Tatsumi K., Takeda S., Konishi I. (2016). Simvastatin inhibits oxidative stress via the activation of nuclear factor erythroid 2-related factor 2 signaling in trophoblast cells. J. Obstet. Gynaecol. Res..

[163] Zhang Y., Liang B., Meng F., Li H. (2021). Effects of Nrf-2 expression in trophoblast cells and vascular endothelial cells in preeclampsia. Am. J. Transl. Res..

